# Functionalization of Polycaprolactone Electrospun Osteoplastic Scaffolds with Fluorapatite and Hydroxyapatite Nanoparticles: Biocompatibility Comparison of Human Versus Mouse Mesenchymal Stem Cells

**DOI:** 10.3390/ma14061333

**Published:** 2021-03-10

**Authors:** Khrystyna Malysheva, Konrad Kwaśniak, Iaroslav Gnilitskyi, Adriana Barylyak, Viktor Zinchenko, Amir Fahmi, Olexandr Korchynskyi, Yaroslav Bobitski

**Affiliations:** 1 Department of Human Immunology, Faculty of Medicine, University of Rzeszow, Warzywna 1A, 35-959 Rzeszow, Poland; khrystyna.malysheva@gmail.com (K.M.); konkwasniak@gmail.com (K.K.); 2 Centre for Innovative Research in Medical and Natural Sciences, Faculty of Medicine, University of Rzeszow, Warzywna 1A, 35-959 Rzeszow, Poland; 3 “NoviNano Lab” LLC, Pasternaka 5, 79015 Lviv, Ukraine; iaroslav.gnilitskyi@novinano.com; 4 Department of Photonics, Lviv Polytechnic National University, S. Bandera 12, 79013 Lviv, Ukraine; yaroslav.v.bobytskyi@lpnu.ua; 5 Department of Therapeutic Dentistry, Danylo Halytsky Lviv National Medical University, Pekarska 69b, 79010 Lviv, Ukraine; adriana.barylyak5@gmail.com; 6 Department of Chemistry of Functional Inorganic Materials, Bogatsky Physico-Chemical Institute of the National Academy of Sciences of Ukraine, Lustdorfska doroga 86, 65080 Odessa, Ukraine; vfzinchenko@ukr.net; 7 Faculty of Technology and Bionics, Rhine-Waal University of Applied Science, Marie-Curie 1, 47533 Kleve, Germany; Amir.Fahmi@hochschule-rhein-waal.de; 8 Department of Biotechnology and Radiology, S.Gzhytskyi National University of Veterinary Medicine and Biotechnologies, 79010 Lviv, Ukraine; 9 Department of Molecular Immunology, Palladin Institute of Biochemistry of the National Academy of Sciences of Ukraine, 01161 Kyiv, Ukraine; 10 Institute of Physics, Centrum of Microelectronics and Nanotechnology, University of Rzeszow, S. Pigonia 1, 35-959 Rzeszow, Poland

**Keywords:** polycaprolactone based scaffolds, hydroxyapatite, fluorapatite, electrospinning, mesenchymal stem cells

## Abstract

A capability for effective tissue reparation is a living requirement for all multicellular organisms. Bone exits as a precisely orchestrated balance of bioactivities of bone forming osteoblasts and bone resorbing osteoclasts. The main feature of osteoblasts is their capability to produce massive extracellular matrix enriched with calcium phosphate minerals. Hydroxyapatite and its composites represent the most common form of bone mineral providing mechanical strength and significant osteoinductive properties. Herein, hydroxyapatite and fluorapatite functionalized composite scaffolds based on electrospun polycaprolactone have been successfully fabricated. Physicochemical properties, biocompatibility and osteoinductivity of generated matrices have been validated. Both the hydroxyapatite and fluorapatite containing polycaprolactone composite scaffolds demonstrated good biocompatibility towards mesenchymal stem cells. Moreover, the presence of both hydroxyapatite and fluorapatite nanoparticles increased scaffolds’ wettability. Furthermore, incorporation of fluorapatite nanoparticles enhanced the ability of the composite scaffolds to interact and support the mesenchymal stem cells attachment to their surfaces as compared to hydroxyapatite enriched composite scaffolds. The study of osteoinductive properties showed the capacity of fluorapatite and hydroxyapatite containing composite scaffolds to potentiate the stimulation of early stages of mesenchymal stem cells’ osteoblast differentiation. Therefore, polycaprolactone based composite scaffolds functionalized with fluorapatite nanoparticles generates a promising platform for future bone tissue engineering applications.

## 1. Introduction

Injuries and diseases associated with bones still represent an important clinical challenge [1,2,3]. Although the bone has a certain ability to self-repair and regrowth, its self-regeneration capacity is still limited [4,5,6,7]. Large bone defects or injuries, caused by complex trauma (including military and traffic accident), non-jointing fractures, bone tumor resection, arthroplasty surgery, infection or genetic disorders are serious problems in orthopedics and dentistry [3,8,9,10]. Clinically, this can lead to non-union of bone and the loss of functional support to surrounding tissues, with the consequence of significant impact on the quality of patients life [3,10]. Moreover, as the bone regenerative capability decreases with age, the incidence of bone diseases and conditions increases dramatically, especially in populations where aging is coupled with increased obesity and poor physical activity [10,11]. Therefore, efficient high-quality bone grafting is crucially important for the healing of the bone defects that occur as a result.

Autologous bone grafting is still regarded as the “gold standard” for bone defects healing. In spite of that, complications and limited availability combined with its harvesting process like secondary damages, high donor site morbidity, expensiveness, limitation of special shape, etc., restrain the use of autologous bone graft in bone tissue regeneration [3,12]. On the other hand, allografts and xenografts can be a solution but face difficulties such as rejection by the immune system and/or potential transmission of infectious or prion diseases [3,10]. Thus, given such limitations inherent with traditional strategies in bone grafting, tissue engineering presents itself as a promising approach for bone repair and regeneration [10].

The biomechanical system of bone is complex so the scaffolds for tissue engineering should follow several key requirements: biocompatibility, which describes scaffolds’ ability to support normal cellular activity as well as lack of toxicity and inflammatory reactions and biodegradability [13,14,15]. Ideally, biomimetic scaffolds must be biodegradable, to allow cells to produce their own extracellular matrix for programmed safe substitution of the scaffold material with osteoid deposition [14,16,17,18]. An ideal bone scaffold must also possess both osteoconductivity, i.e., the ability of scaffolds to provide inward migration of osteoinducible cellular elements such as mesenchymal cells, osteoblasts and osteoclasts, and osteoinductivity which refers to inducing the differentiation of cells from different lineages into osteogenic cells [14,19,20]. Moreover, scaffolds for bone tissue regeneration must possess appropriate mechanical properties which should match the bone properties and pore size similar to the host for successful blood vessels formation, diffusion of essential nutrients and oxygen for cell survivability [13,21].

Different types of biomaterials which are basic components of scaffolds have been used in bone tissue engineering [22]. Natural biomaterials have many advantages. However, they are difficult to engineer due to their limited processing ability, high risk of contamination and batch-to-batch variability [23]. These concerns led to the exploration of synthetic biodegradable polymers for use in biomedical applications [24]. Synthetic polymers are highly popular as scaffold material, as they possess defined chemistry with reasonable processing and tailoring ability and can be modified to achieve desired properties for specific applications. Other merits include cost efficacy, ability to be produced in large quantities uniformly and a longer shelf time. In addition, their physicochemical and mechanical properties such as tensile strength, elastic modulus and degradation rate are comparable to bone [9,23,25].

Within the class of synthetic biomaterials, poly ε-caprolactone (PCL) has recently drawn significant attention for biomedical applications including bone tissue engineering [23,24,26,27]. PCL is a semicrystalline (50%) linear aliphatic polyester with several desirable features [24,28,29]. It possesses good stability under ambient conditions, ease of processability (thermal and solution) and superior mechanical properties (high strength, elasticity depending on its molecular weight) [24,28,29]. PCL is also tissue compatible, bioresorbable and biodegradable biomaterial that produces promising results in clinical use [23,24,30,31,32]. However, its low bioactivity, hydrophobic behavior and long term degradation in vivo may limit applications for pure PCL [33,34]. At the same time, all the advantages of PLC matrix make it a highly promising therapeutic platform for multiple modifications. Electrospun PLC nanofibers already were tested as a scaffold for generation of biocompatible supermagnetic nanofibrous, carbon nanotubes or mesoporous silica coating [35,36,37]. Mentioned composite biomaterials were successfully tested for osteoinductive and osteoconductive properties with rat [35,36], or human [37] bone marrow-derived mesenchymal stem cells.

Another class of composite biomaterials for bone repair include ceramics such as hydroxyapatite (HAp) [38,39,40] and fluorapatite (FAp) [17,22]. HAp is one of the most widely clinically used biomaterial in bone tissue engineering. It has been considered to be the ideal material to create bone tissue engineering scaffolds due to its structures’ close resemblance to nature bone mineral, great biocompatibility, osteoconductivity and osteoinductive properties. HAp products have been used in dental and orthopedic surgeries to fill in bone defects and to coat metallic implant surfaces to improve implant integration with the host bone. However, HAp scaffolds show high brittleness, poor mechanical stability and difficulty of shaping, all of which greatly limit its application [39,41,42,43]. Pure FAp is another bioactive and biocompatible ceramic [44,45], that is structurally and chemically similar to HAp but it is known to be more chemically stable and easier to synthesize [46,47]. In addition, it has a much lower solubility in biological fluids than HAp. In several studies, it has been proved that the amount of the released fluoride ions affects directly the cell attachment, proliferation, morphology and differentiation of osteoblast cells [47,48,49]. Moreover, FAp increases the bone density and acts as an anti-carcinogenic agent [47,48,50,51,52]. It is also known to possess a potential advantage with its high chemical stability and aptitude to delay caries’ process without the biocompatibility reduction [46,47].

To solve challenges associated with the mentioned biomaterials, polymer/ceramic composite scaffolds have been fabricated. The use of electrospinning for the fabrication of the scaffolds facilitates manipulation of the morphology and offers control over the macro- and microstructure of the scaffolds. In addition, the polymers used for electrospinning act as the porous matrix for the ceramics to reduce the brittleness, while providing a much larger contact surface area [13,39,42,43].

In a view of the above-mentioned advantages, the main goal of the research is to fabricate composite scaffolds for bone tissue regeneration which will comply with all key requirements listed above. In this paper, a description of the synthesis and the characterization of PCL based composite scaffolds enriched with different amounts of HAp and FAp nanoparticles has been provided. The comparative study of their physicochemical properties, in vitro biocompatibility and osteoinductivity in mouse and human preosteoblast models has been performed using various physical and biological methods.

## 2. Materials and Methods

### 2.1. Materials and Fabrication of PCL/HAp and PCL/FAp Composite Scaffolds

#### 2.1.1. Synthesis of Apatites (HAp and FAp) in Saline Melts

Nanoparticles of HAp (Ca_10_(PO_4_)_6_(OH)_2_) and FAp (Ca_10_(PO_4_)_6_F_2_) were synthesized using low-temperature synthesis method in a saline melt that was proposed by Nechyporenko and Zinchenko [53]. Briefly, synthesis was carried out in muffle furnace at temperature 350 °C in a saline melt of eutectics KNO_3_-NaNO_3_ during 2 h [53,54]. Interaction occurs on following schemes:

6KPO_3_ + 10Ca(OH)_2_ → Ca_10_(PO_4_)_6_(OH)_2_ + 6KOH + 6H_2_O↑ 6Na_3_PO_4_ + CaF_2_ → Ca_10_(PO_4_)_6_F_2_ + 18NaF (1)

The phase structure and lattice parameters of the obtained apatites were described in detail previously [53].

#### 2.1.2. Electrospinning

The program for electrospinning was set up using the parameters mentioned in Table 1.

### 2.2. Characterization of PCL/HAp and PCL/FAp Composite Scaffolds

#### 2.2.1. Scanning Electron Microscopy

Nanofibers morphology of PCL/HAp and PCL/FAp composite scaffolds was examined using Tescan Vega 3 scanning electron microscope (SEM) supplied by Tescan Analytics (Fuveau, France). Prior to the study, the scaffolds were covered with a conductive layer of gold by sputter coater Cressington 108 Serie Sputter Coater. The analysis was performed in high-vacuum mode at 30.0 kV. Three samples of each obtained scaffold were analyzed by using the ImageJ^®^ software to analyze the average diameter of the nanofibers.

#### 2.2.2. Static Contact Angle Determination

The contact angle of PCL/HAp and PCL/FAp scaffolds’ surfaces were measured at RT (22 °C) using Theta Flex optical tensiometer supplied by Biolin Scientific (Västra Frölunda, Sweden). The water contact angle of the 7 samples was evaluated by static contact angle measurements using the sessile drop method. In general, a 1 µL droplet of distilled water was placed on the surface of studied dry scaffolds and the image of the drop was recorded for 10 s. An average value of contact angle was calculated on the basis of at least five measurements. Static contact angle was then defined by fitting Young-Laplace equation around the droplet.

### 2.3. In Vitro Biocompatibility Study of PCL/HAp and PCL/FAp Composite Scaffolds

#### 2.3.1. Scaffold Sterilization and Preparation for Cell Culture

Before sterilization, PCL, PCL/HAp and PCL/FAp scaffolds’ mats were cut into square specimens of 3 mm × 3 mm or 5 mm × 5 mm (depending on the type of experiment). Thereafter, studied scaffolds underwent two steps of sterilization process: immersion in 70% (*v*/*v*) ethanol/water solution for 30 min and sterilization under a UV light for 30 min for each side of the scaffolds’ mat.

After sterilization and prior to seeding cells, scaffolds’ mats were pre-incubated in complete cell culture medium supplemented with 10% fetal bovine serum (FBS, Biowest, Nuaillé, France) for overnight in incubator. Further, the medium was removed and 10 or 20 μL of cellular suspension was placed on the top of mat. Prior to adding remaining culture medium to each well, plates containing cell-loaded scaffolds were incubated under standard cell culture condition for 1 h to allow cells to adhere to the scaffolds.

In order to prepare the matrices’ extracts, the sterile PCL, PCL/HAp and PCL/FAp scaffolds’ samples were incubated at ratio 1:100 (*w*/*v*) in previously prepared Dulbecco’s modified Eagle’s medium (DMEM, Biowest, Nuaillé, France) supplemented with 10% FBS and 1% penicillin/streptomycin (Biowest, Nuaillé, France) for 24 h at 37 °C under continuous steering. Then, scaffolds’ samples were incubated while being immersed into the same medium for one week at 4 °C. The procedure was repeated to improve the efficiency of the extraction process and two portions of each matrix extract were pulled together. The obtained sample incubation medium (scaffold extract) was used for cytotoxicity study.

#### 2.3.2. Cell Culture

The following cell lines were used in this study: immortalized mouse mesenchymal stem cells of C2C12 line and human mesenchymal stem cells (hMSC). C2C12 cells were purchased from American Type Culture Collection (ATCC, Manassas, VA, USA) and hMSC were isolated from bone marrow aspirate of the surgical material obtained upon hip replacement surgery under informed patient’s consent. The hMSC multipotency was estimated based on their capacity to efficiently differentiate into osteoblast, adipocyte and chondrocyte lineages upon proper stimulation and conditions. C2C12 cells were cultivated in high-glucose DMEM containing 10% FBS and 1% penicillin/streptomycin. hMSC were maintained in a growth medium consisting of low-glucose DMEM supplemented with 10% FBS, 1% penicillin/streptomycin and 10 ng/mL of basic fibroblast growth factor (Peprotech, Rocky Hill, NJ, USA). All cultures were incubated at a 5% CO_2_-containing atmosphere at 37 °C and 95% humidity. Culture medium was refreshed every 2–3 days.

#### 2.3.3. Cell Viability Assay

3-(4,5-Dimethylthiazol-2-yl)-2,5-diphenyltetrazolium bromide or MTT assay was used for assessing cell metabolic activity as an indicator of cell viability [55,56]. The assay reflected the activity of a mitochondrial dehydrogenase that transforms light yellow MTT into purple Formosan. In this regard, C2C12 cells and hMSCs were seeded at a density 2500 and 5000 cells/well containing 100 µL of culture medium in 96-well plates, respectively. To allow attachment cells were cultured for 24 h and then culture medium was replaced with appropriate 100% scaffolds’ extracts and further cultivated for additional 72 h. Then, the MTT assay of viable cells was performed in accordance with the manufacturer’s recommendations (Sigma Aldrich/Merck, Steinheim, Germany). The reaction product was quantitatively determined by an Infinite 200 PRO microplate reader purchased from Tecan (Männedorf, Switzerland) at a wavelength of 570 nm. The wells without the extracts and the free cells (culture medium alone) were used as blanks.

#### 2.3.4. DAPI Staining

4′,6-Diamidino-2-phenylindole (DAPI) staining was used to evaluate cell attachment to PCL/HAp and PCL/FAp scaffolds’ mats. C2C12 cells and hMSCs were seeded at a density 5000 cells/scaffold in 24-well plates. The medium was changed every two days. After 1 week of cultivation, C2C12 cells and hMSCs were washed twice with phosphate-buffered saline or PBS, (EURx, Gdańsk, Poland) and then fixed with 4% paraformaldehyde (Alfa Aesar/Thermo Fisher Scientific, Tewksbury, MA, USA) for 10 min. After that, cells were washed twice with PBS and permeabilized with 0.1% Triton X-100 (Sigma Aldrich/Merck, Steinheim, Germany) for 10 min, washed again twice with PBS and then stained with PureBlue DAPI Nuclear Staining Dye (BioRad, Irvine, CA, USA) for 15 min. After staining, cells were washed three times with PBS and evaluated by a fluorescent microscopy using Axio Observer Z1 inverted phase contrast fluorescence microscope purchased from ZEISS (Oberkochen, Germany).

#### 2.3.5. Induction of Osteoblast Differentiation

For osteogenic studies C2C12 cells were seeded on PCL/HAp and PCL/FAp scaffolds’ mats in 96- or 24-well plates at a density of 10,000 or 20,000 cells/scaffold, respectively. Following cellular attachment, the growth medium was replaced by osteogenic differentiation medium supplemented with 10 ng/mL of recombinant BMP2/7 (R&D Systems, Minneapolis, MN, USA) and 50 µg/mL of ascorbic acid (Sigma Aldrich/Merck, Steinheim, Germany). Cells were cultured for 4 days prior to differentiation assessment.

#### 2.3.6. Alkaline Phosphatase Assay

The alkaline phosphatase (ALP) activity produced by C2C12 cells was analyzed spectrophotometrically using a π-nitrophenylphosphate (π-NPP) as a substrate [57]. Four days after induction of osteoblast differentiation, the cells were washed twice with PBS, lysed in ALP lysis buffer (10 mM glycine, 100 µM MgCl2, 10 µM ZnCl2 and 0.1% Triton X-100) and agitated gently for 5 min. Then, 10 µL aliquot of cell lysate was placed into a 96-well plate and ALP activity was revealed with 90 µL/well of ALP assay buffer (100 mM glycine, 1 mM MgCl_2_ and 100 µM ZnCl_2_) supplemented with 6 mM π-NPP (Thermo Fisher Scientific, Waltham, MA, USA) [57]. Samples were mixed gently and incubated at RT until color developed (ALP converts the π-NPP substrate to an equal amount of colored p-nitrophenol (pNP)). Colorimetric absorbance was measured at a wavelength of 405 nm using an Infinite 200 PRO microplate reader (Tecan, Männedorf, Switzerland). The level of ALP expression is directly proportional to the intensity of early osteoblast differentiation of C2C12 cells [57].

#### 2.3.7. Alkaline Phosphatase (Histochemical) Staining

Extracellular ALP was qualitatively evaluated by histochemical staining at day 4 after induction of osteoblast differentiation. For such purpose, C2C12 cells were washed twice with PBS and subsequently fixed with 4% paraformaldehyde at RT for 5 min. The fixed cells were further washed twice with PBS and stained with a solution containing Fast Blue RR salt (Sigma Aldrich/Merck, Steinheim, Germany) and Naphthol AS-MX phosphates alkaline solution (Sigma Aldrich/Merck, Steinheim, Germany) at RT for 2 h. After discarding the staining solution and washing the plates with PBS, the cells were observed using Primovert inverted phase contrast microscope (ZEISS, Oberkochen, Germany). The resulting blue, insoluble, granular dye deposit indicates sites and the intensity of ALP activity.

#### 2.3.8. Statistical Analysis

All experiments were repeated twice in duplicates (osteogenesis assays) or triplicates (cellular viability and contact angle assays). Data were analyzed using GraphPad Prism 6 (Graphpad Software, San Diego, CA, USA). Results of MTT assay and spectrophotometric measurements of ALP activity are represented as mean ± standard deviation (SD). Statistical differences between experimental variants were assessed by Student’s *t*-test and the *p* value of less than 0.05 was considered significant.

## 3. Results and Discussion

### 3.1. Morphological Structure

**Figure 1:**
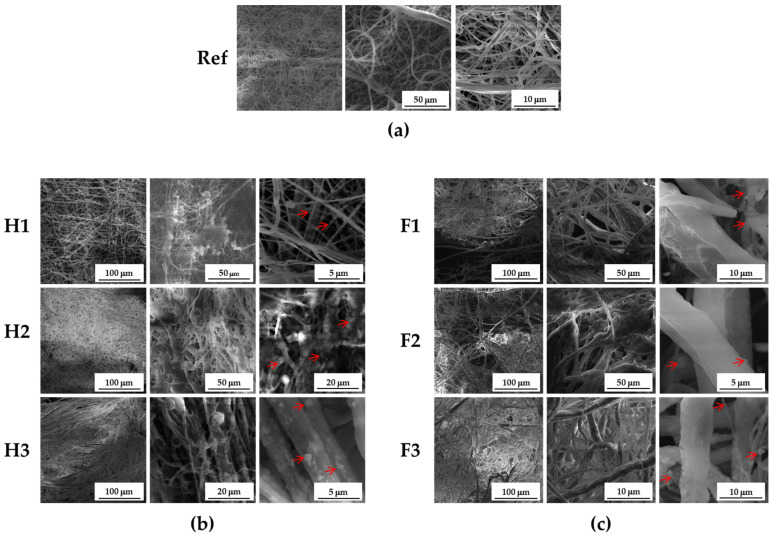
SEM images of electrospun nanofibers: (**a**) pure PCL and its blends containing (**b**) 1% (H1), 3% (H2) and 7% (H3) HAp or (**c**) 1% (F1), 3% (F2) and 7% (F3) FAp, respectively. Representative images are shown at different magnification. Red arrows indicate the aggregates of nanophased HAp or FAp deposited onto the nanofiber surface.

The morphological structure of fabricated scaffolds is an important design consideration in bone tissue engineering [58]. It is known, that fiber morphologies influence the cell attachment and facilitate control over the cell behavior [58]. To examine the microstructure of the obtained electrospun PCL/HAp and PCL/FAp composite scaffolds, the samples were carefully cut and studied with scanning electron microscopy. The following SEM images (Figure 1) demonstrate an average thickness of fibers within the scaffolds varying in the range of 2–10 micrometers.

The fibers seem to have large deviation of average diameter; however, smooth fibers without the presence of beads were obtained. The presence of functional agents in the electrospun fibers decreased their uniformity in case of FAp, which is much more prevalent with the increasing concentration of FAp. However, the fibers obtained with HAp as a functional agent appear to be uniform. Moreover, they exhibit an absence of coagulated fibrils structure which is evident with functionalization with FAp, especially at higher concentrations. This is presumably due to the lower solubility of FAp in ethanol as compared to HAp in electrospinning suspension, thus providing lesser control over the entire electrospinning process. The functionalized fibers possess dimpled structures on their surfaces due to the presence of the apatite particles which are absent in pristine fibers. Additionally, a great variance of size dispersion of FAp and HAp particles significantly influenced the stability of electrospinning suspension which is evident from the scanning electron microscopy results. Therefore, by controlling the size dispersion of particles and tweaking the electrospinning parameters, it should be possible to obtain fibers with even lower dimensions and a much greater control over the average diameter of fibers.

### 3.2. Contact Angle (Wetting Angle)

**Figure 2:**
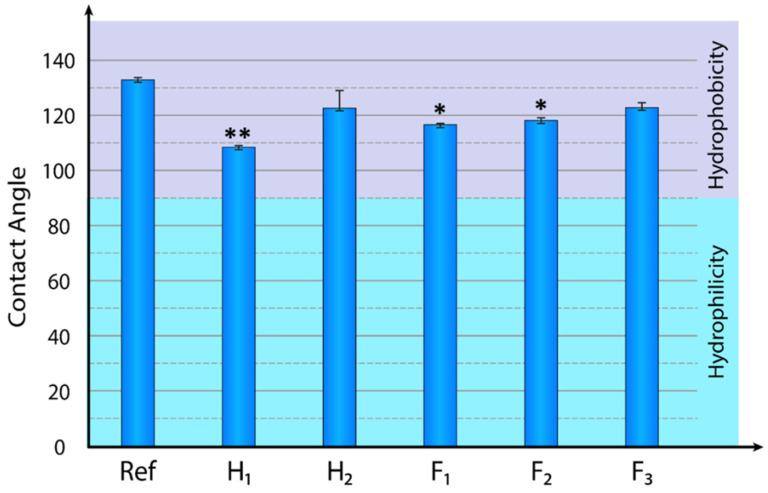
Measurement of static contact angle of a water droplet on a solid surface of electrospun nanofibers of pure PCL spotted as Ref and its blends containing HAp, noted as H1—1% (*w*/*v*) and H2—3% (*w*/*v*); FAp noted as F1—1% (*w*/*v*), F2—3% (*w*/*v*), F3—7% (*w*/*v*), respectively. Data are presented as mean ± SD (*n* = 5). *p*-values were calculated based on *t*-test. Asterisks show the significance levels of difference for particular variants versus Ref (pure PLC): ** for *p* < 0.01; * for *p* < 0.05.

The surface wettability of biomaterials plays an important role for the attachment and proliferation of different cells [59,60,61]. It is known that changes in the wettability properties of the materials may result from chemical or physical modifications of the surfaces. Hydrophilicity (wetting) and hydrophobicity (non-wetting) are demonstrated by the con-tact angle below or above 90°, respectively. To investigate the surface properties of pure PCL and PCL scaffolds enriched with HAp or FAp nanoparticles the static contact angle was estimated using the sessile drop method. Distilled water drops (1 μL) at a controlled minimum mean deviation were placed on the surface of studied nanofibers mats. Figure 2 summarizes the contact angle measurement performed on pure PCL and PCL scaffolds modified at the different proportions with HAp or FAp nanoparticles, respectively.

For pristine (control) PCL fibers depicted on the Figure 2 as Ref, the average wetting angle was within 133°. While for PCL scaffolds enriched with HAp nanoparticles, marked as H1 and H2 the average wetting angles were slightly lower comparably to Ref, 108° and 123°, respectively. The similar behavior was observed also for PCL scaffolds enriched with FAp nanoparticles, where the contact angles slightly below the control one. So, as shown on the Figure 2, the contact angle of F1 is equal to 117° while for F2 and F3 are 121° and 123°, respectively. At the same time, in spite of a high negative charge of fluoride ion, the difference in hydrophobicity between 1% HAp and 1% FAp as well as 2% HAp and 2% FAp appeared to be non-significant (Figure 2).

The PCL due to its hydrophobic nature provides strong contact angle which is even more effective after electrospinning in the form of fibers. It can be regarded as a non-wetting surface with weak solid phase–liquid interaction and strong liquid–liquid interaction. Furthermore, it is evident from experimental studies that the wettability of PCL scaffold containing HAp nanoparticles slightly decreases and that the lower the concentration of Hap, the higher the wettability. This could be attributed to increase surface area of the fibers due to emergence of dotted submicron structures on the surface in the presence of HAp.

Therefore, the wetting angle of FAp nanoparticles-containing PCL scaffolds shows quite similar properties to HAp nanoparticles. This observation supports the conclusion that the presence of both FAp and Hap nanoparticles provides hydrophilic properties to the scaffold, in such a way as to overcome the inherent hydrophobic nature of the PCL polymer used for fabrication of fibers. It should be noted that the dynamics of wetting properties was almost negligible within the studied concentrations of apatites.

### 3.3. In Vitro Cytocompatibility

**Figure 3:**
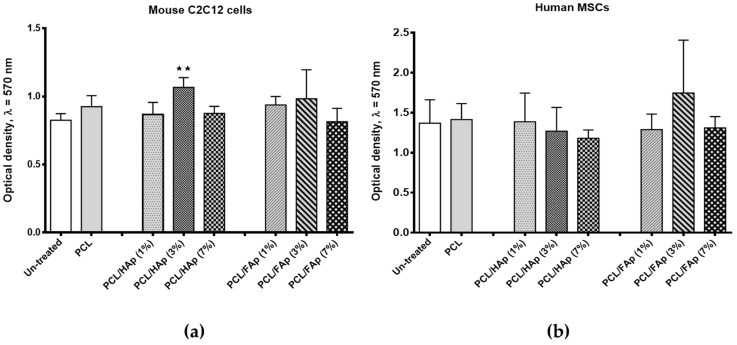
The effect of PCL/HAp and PCL/FAp scaffold extracts on the in vitro growth of (**a**) mouse C2C12 cells and (**b**) human MSCs measured using MTT assay. Data are presented as mean ± SD (*n* = 3). *p*-values were calculated based on the *t*-test. Asterisks show the significance levels of difference for particular variants versus un-treated cells (** for *p* < 0.01).

MTT chromometry assay was used to estimate the potential cytotoxicity of fabricated PCL scaffolds with different concentrations of HAp or FAp towards our experimental models: mouse mesenchymal precursor cells of C2C12 line and human bone marrow derived hMSCs. 100% extracts from PCL scaffolds containing 1%, 3% and 7% of HAp or FAp were added to cells for 72 h. The cells cultivated without scaffold extracts or with 100% extracts from pure PCL scaffold were used as the negative controls. The MTT assay results of C2C12 cells and hMSCs viability determination are shown on Figure 3a,b.

After 72 h of C2C12 cells incubation with scaffold extracts of PCL containing 3% of HAp and 3% of FAp, we observed a slight increase in cell proliferation, when compared with controls. In the case of scaffolds extracts of PCL containing 1% and 7% of HAp or FAp, the result was similar to that observed for un-treated cells and cells incubated with pure PCL scaffold extract. At the same time, C2C12 cells proliferation was significantly higher (*p* < 0.01) after treatment with PCL containing 3% of HAp in comparison with un-treated cells. The treatment of human MSCs with PCL/HAp or PCL/FAp scaffold extracts did not substantially affect their growth in comparison with the controls. Hence, we observed an insignificant decrease in cell proliferation after incubation with scaffold extract of PCL containing 7% of HAp and a slight non-significant increase of hMSCs growth after treatment with scaffold extract of PCL containing 3% of FAp. Thus, performed in vitro cell studies revealed good cytocompatibility of the obtained PCL/HAp and PCL/FAp scaffolds.

### 3.4. Cellular Attachment

**Figure 4:**
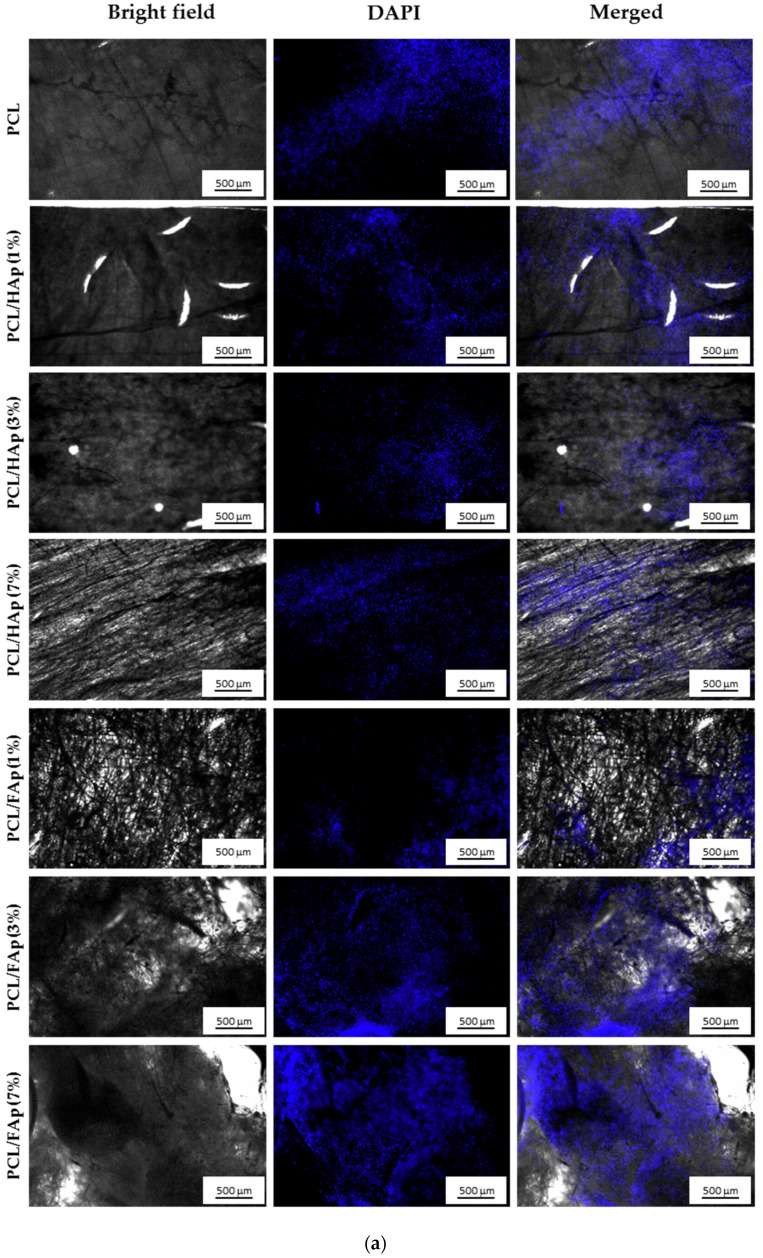

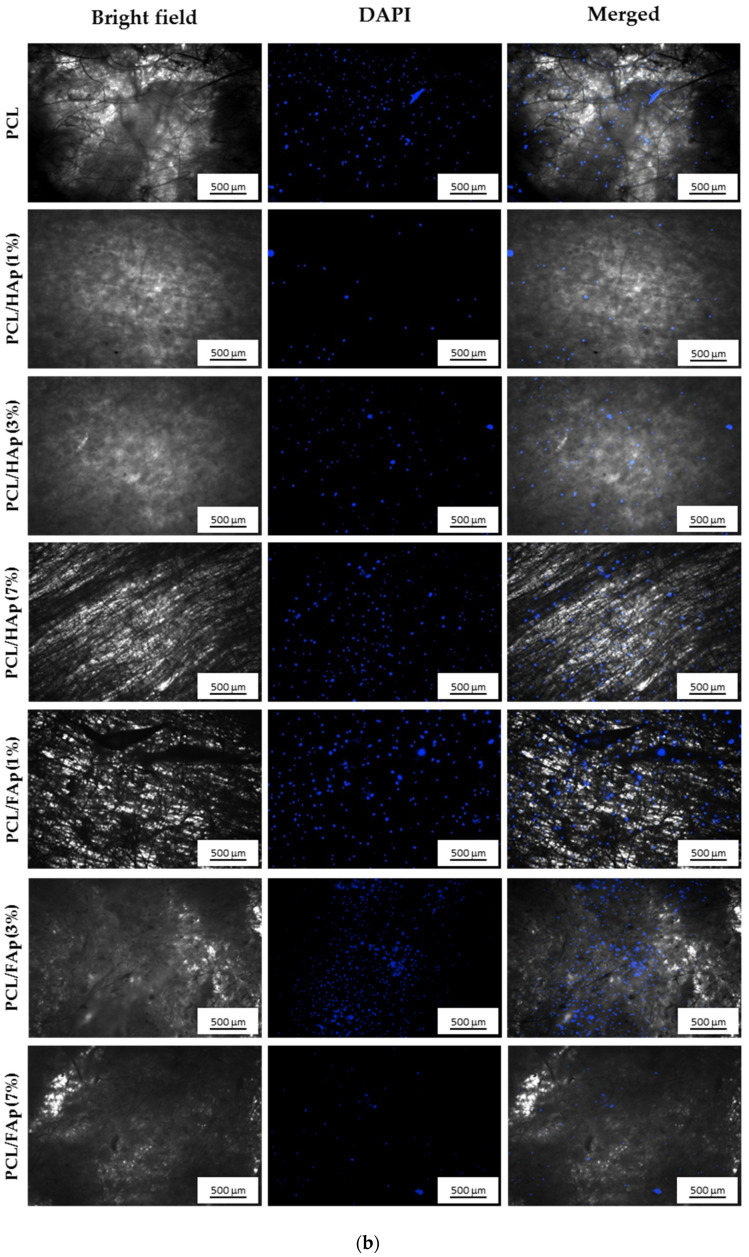
(**a**) Fluorescence microscope images for DAPI staining (blue nucleus) of mouse C2C12 cells seeded onto the pure PCL and PCL scaffolds containing 1%, 3% and 7% of HAp or FAp after 7 days of culture. Representative images are shown at 5× magnification and scale bars at 500 μm. (**b**) Fluorescence microscope images for DAPI staining (blue nucleus) of human MSCs seeded onto the pure PCL and PCL scaffolds containing 1%, 3% and 7% of HAp or FAp after 7 days of culture. Representative images are shown at 5× magnification and scale bars at 500 μm.

Mouse C2C12 cells and human MSCs attachment to the surfaces of pure PCL and PCL scaffolds containing 1%, 3% and 7% of HAp or FAp was evaluated using DAPI staining. Figure 4a,b show DAPI labeled cells visualized under the fluorescence microscope. After 7 days of culturing, we observed difference in attached C2C12 cells or hMSCs seeded on the pure PCL, used as a control and studied composite PCL scaffolds. In general, cellular distribution was different depending on the HAp and FAp concentration in scaffolds and its network architecture.

As shown on Figure 4a, the pure PLC scaffold appeared to be a good substrate for C2C12 cells attachment. In spite of well-known intrinsic osteoinductive properties of HAp, composite PLC-HAp matrices were less suitable for C2C12 cells attachment and growth. PCL-based scaffolds containing 3% and 7% of FAp visually had the highest numbers of mouse C2C12 cells while all other studied scaffolds showed comparatively similar and lower cell numbers.

Interestingly, in the case of human MSCs (Figure 4b), both pure PLC and PLC-HAp composites of low HAp content (1 and 3%) appeared to be inefficient substrates for cellular attachment. The highest numbers of cells were observed on PCL scaffolds containing 7% of HAp and 1% and 3% of FAp in comparison with other HAp- or FAp-containing scaffolds.

Thus, incorporation of FAp nanoparticles enhances the ability of the composite scaffolds to interact and support the human bone marrow derived cells on their surfaces in comparison with HAp containing PCL scaffold. At the same time, higher FAp content (7%) into the electrospun bone regenerative matrix starts to reduce the hMSC cellular attachment and/or growth. Such phenomenon resembles the situation with a tooth enamel: deficiency of fluoride into a diet reduces its strength and increases the risk of injury by carries. On the other hand, the chronic excessive fluoride into a diet leads to endemic dental fluorosis with the significant hypomineralization of tooth enamel [62,63], thus, also resulting in a reduced enamel strength.

### 3.5. Early Osteoblast Differentiation

ALP expression was used for the assessment of the effect of PCL scaffolds containing 1%, 3% and 7% of HAp or FAp on the early stages of C2C12 cells osteogenic differentiation.

**Figure 5:**
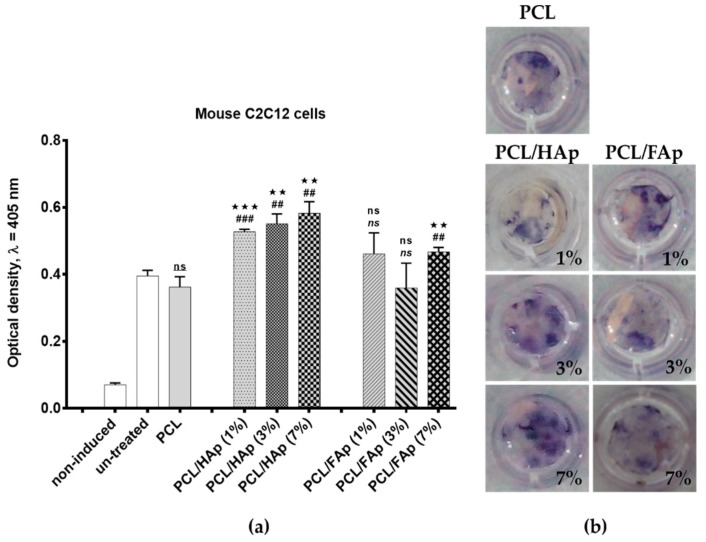
Early BMP2/7-induced osteoblast differentiation of C2C12 cells evaluated on ALP activity after 4 days of culturing on pure PCL and PCL scaffolds containing 1%, 3% and 7% of HAp or FAp. Data of (**a**) spectrophotometric measurement of ALP activity are presented as mean ± SD (*n* = 3); *p*-values were calculated based on *t*-test. Asterisks show the significance levels of difference for particular variants versus controls: **(##) for *p* < 0.01; ***(###) for *p* < 0.001; ns (ns, ns)—non-significant. Notes: *—particular variants versus cells cultured on pure PCL scaffold; #—particular variants versus cells cultured without scaffold; ns—particular variants versus cells cultured on pure PCL scaffold; ns—particular variants versus cells cultured without scaffold; ns—cells cultured on pure PCL scaffold versus cells cultured without scaffold. Representative fields of (**b**) histochemical staining of extracellular ALP is shown at 1× magnification.

As is shown on Figure 5a, spectrophotometric measurement of ALP activity clearly proved the activation of C2C12 osteoblast differentiation in all studied samples after 4 days of culturing. Mouse C2C12 cells cultured on pure PCL scaffold and/or C2C12 cells cultured without scaffold were used as controls.

In particular, we observed significantly higher ALP activity in cells cultured on PCL scaffolds containing 1% (*p* < 0.001), 3% (*p* < 0.01) and 7% (*p* < 0.01) of HAp, whereas ALP activity in cells cultured on PCL scaffolds containing 1% and 3% of FAp was comparable with controls. The ALP activity in cells cultured on PCL scaffolds containing 7% was also significantly higher in comparison with controls, but at the same time, lower than those containing HAp nanoparticles.

Figure 5b qualitatively demonstrates histochemical ALP staining of C2C12 cells on studied scaffolds at day 4. It is obvious that ALP staining on the PCL scaffold containing 7% HAp is stronger than ALP staining on the other HAp and FAp containing PCL scaffolds and control pure PCL. In contrast, ALP staining on the PCL scaffold containing 3% and 7% of FAp was weaker than those cultured on the PCL scaffold containing 1% of FAp or on other studied scaffolds. 

The main disadvantage of modern material science lays in the usual absence of systematic approach to the evaluation of graft biomaterials features. In our studies, we tried not only to compare two composite materials at the different concentrations of incorporated nanoparticles, but also attempted to demonstrate in the same study the difference between the behavior of mesenchymal stem cells of human and mouse origin. It is very intriguing that in spite of very similar physico-chemical properties of FAp and Hap materials, only human mesenchymal stem cells demonstrate the preference to FAp-enriched matrix and such their behavior is reminiscent to requirement of precise levels of fluoride observed into dental hygiene. Such a systematic approach would be very beneficial with further developing to in vivo studies. Previously available experimental data did not provide any mechanistical clue for the requirement of precise fluoride levels with the adverse effects for facial bone homeostasis observed for both deficit and excess of fluoride. Our results suggest that only intermediate levels of fluoride (3% FAp in our experiments) incorporated into osteoplastic material might be beneficial for optimal recruitment and osteogenesis by human MSC.

## 4. Conclusions

In the current paper, we described physicochemical and biological properties of novel PCL-based electrospun osteoplastic scaffolds modified with FAp or HAp nanoparticles. The mentioned ceramics have been used to improve the osteoconductive and osteoinductive properties of PCL scaffold to be used as the grafts for bone tissue regeneration. The studied composite scaffolds containing different amounts of FAp or HAp (1%, 3% and 7%) nanoparticles were successfully fabricated through the electrospinning process. The measurement of static contact angle of pure PCL scaffold versus PCL/HAp and PCL/FAp composite scaffolds showed that the presence of HAp and FAp nanoparticles slightly decreases hydrophobic properties of the scaffold and respectively improve its wettability. Furthermore, in vitro cell studies of osteoconductivity revealed non-toxicity and good cytocompatibility of both the obtained PCL/HAp and PCL/FAp composite scaffolds. Moreover, incorporation of FAp nanoparticles enhances the ability of the scaffolds to interact and support the mouse and human MSCs on their surfaces compared to HAp containing PCL scaffolds. On the other hand, the investigation of osteoinductive properties of PCL/HAp and PCL/FAp composite scaffolds demonstrated the increased similar ability of HAp (7%) and FAp (1%) to stimulate early stages of osteoblasts’ differentiation.

Thus, the performed studies indicate that incorporation of PCL/FAp composite into a PLC scaffold has improved its wettability and also has a potential to improve osteoinductive and osteoconductive properties of matrix. Therefore, it is a suitable platform to be proposed as a biomaterial composite for possible application in bone tissue engineering, while comprehensive evaluation of its potential still requires appropriate in vivo and clinical studies.

## Figures and Tables

**Table 1:** Description of solutions and parameters used for electrospinning.

Sample	PCL	Functional Agent	Solvents (mL)	Voltage	Flow Rate	Humidity, Temperature
**Reference**	18% (*w*/*v*)	0	DCM (2) + Ethanol (3)	16 kV, −4 kV	0.3 mlh^−1^	80%, 18 °C
**H1**	18% (*w*/*v*)	HAp 1% (*w*/*v*)	DCM (2) + Ethanol (3)	16 kV, −4 kV	0.3 mlh^−1^	80%, 18 °C
**H2**	18% (*w*/*v*)	HAp 3% (*w*/*v*)	DCM (2) + Ethanol (3)	16 kV, −4 kV	0.3 mlh^−1^	80%, 18 °C
**H3**	18% (*w*/*v*)	HAp 7% (*w*/*v*)	DCM (2) + Ethanol (3)	16 kV, −4 kV	0.3 mlh^−1^	80%, 18 °C
**F1**	18% (*w*/*v*)	FAp 1% (*w*/*v*)	DCM (2) + Ethanol (3)	16 kV, −4 kV	0.3 mlh^−1^	80%, 18 °C
**F2**	18% (*w*/*v*)	FAp 3% (*w*/*v*)	DCM (2) + Ethanol (3)	16 kV, −4 kV	0.3 mlh^−1^	80%, 18 °C
**F3**	18% (*w*/*v*)	FAp 7% (*w*/*v*)	DCM (2) + Ethanol (3)	16 kV, −4 kV	0.3 mlh^−1^	80%, 18 °C

## Data Availability

The data presented in this study are available on request from the corresponding author. The data are not publicly due to disclosure agreement.
